# Bedside placement of peritoneal dialysis catheters – a single-center experience from Hungary

**DOI:** 10.1080/0886022X.2019.1614058

**Published:** 2019-06-04

**Authors:** Ákos Pethő, Réka P. Szabó, Mihály Tapolyai, László Rosivall

**Affiliations:** a 1st Department of Internal Medicine, Faculty of Medicine, Semmelweis University, Budapest, Hungary;; b Department of Surgery, Faculty of Medicine, University of Debrecen, Debrecen, Hungary;; c Medical Services, Ralph H. Johnson VA Medical Center, Charleston, SC, USA;; d Hemodialysis Unit, Fresenius Medical Care Hungary, Hatvan, Hungary;; e Department of Pathophysiology, International Nephrology Research and Training Center, Semmelweis University, Budapest, Hungary

**Keywords:** Heart failure, minimally invasive, peritoneal dialysis, percutaneous, PD catheter

## Abstract

**Objectives:** The successful implantation of peritoneal dialysis (PD) catheters is a critical skill procedure with the potential to impact both the short- and long-term success of renal replacement therapy and the patients’ survival.

**Methods:** We retrospectively reviewed our single-center experience with nephrologist-placed minimally invasive, double-cuffed PD catheters (PDCs).

**Results:** The recruitment period was March 2014 through December 2015. The follow-up period lasted until 2016. The mean age of the subjects was 60 ± 18 years and indications for the PD were diuretic resistant acutely decompensated chronic heart failure in seven patients (47%) and end-stage renal disease in eight (53%) patients. Comorbid conditions included diabetes (27%), ischemic heart disease (47%), advanced liver failure (27%), and a history of hypertension (73%). The cohort had a high mortality with five subjects only in severe heart failure group (33%) passing away during the index hospitalization; of the rest, two (13%) had heart transplantation, three (20%) changed modality to hemodialysis, and only five (33%) continued with maintenance PD beyond 1 month. Acute technical complications within the first month were infrequent: one catheter (6%) had drainage problems and one (6%) was lost due to extrusion. There were no serious complications (e.g., organ damage, peritonitis, etc.).

**Conclusions:** In selected cases, particularly in severe diuretic refractory heart failure, PDC placement placed by a nephrologist is feasible with a low rate of complications even in a low-volume center setting. The catheters we placed were all functioning with only minor complications and PD could be started immediately.

## Introduction

Indications and techniques for peritoneal dialysis (PD) as well as the practice of modality will continue to evolve, influenced to a large degree by local needs and the availability of expertise and skills. In Hungary, the modality took root in 1960 [1], with the initial rigid straight catheters gradually replaced by the Tenckhoff catheters in subsequent years [2]. While historically, PD catheters (PDCs) were inserted by surgeons, the need soon became apparent for a simplified procedure utilizing local anesthesia and performed outside of the operating rooms. The fluoroscopic insertion of PDC by nephrologists using the Seldinger technique can be performed in a procedure room, allowing the expeditious initiation of dialysis without involving a surgical team [3]. Those non-surgically inserted PDCs were and still are sutured to the rectus muscle or fascia [4]. Early clinical results confirmed the functional equivalency of non-surgically implanted PDCs to surgically placed ones, also proving to be reliable as long-term PD access [5]. For proper wound healing, a minimum of 4–6-week-long waiting period is needed after PDC placement before starting regular PD with full PD fluid exchange. Further comparative clinical studies had shown that PDC placement is safe even during bedside catheter insertion, particularly for patients with poor cardiac function; it proves to be equally safe when managing secure anesthesia, when endotracheal intubation is impossible, or the surgeon is not comfortable with the procedure in local anesthesia [6–10]. The advantage of minimally invasive catheter insertions is less surgical trauma permitting an earlier start on PD fluid exchange without any waiting period [11–13]. Non-surgical placement is preferable for cases requiring an urgent initiation of dialysis [14]. Long-term retrospective clinical studies had clearly demonstrated the benefits of minimally invasive interventions [15–18] and may offer a viable alternative in resource-limited countries or when the risks of true surgical procedures impede the establishment of modality.

## Materials and methods

This is a retrospective study approved by the Ethics Committee of the Hungarian Health Ministry (equivalent for an Independent Review Board) (TUKEB 28962-3/2018/EKU) and the Fresenius Medical Care as the local dialysis provider in Hungary. The study conforms to the Helsinki Declaration as developed by the World Medical Association. All patients provided written informed consents for the procedure.

The PDC insertion was utilized initially for those with a severe diuretic-resistant acute decompensated heart failure and cardio-renal syndrome; subsequently, it was expanded to other patients with end-stage renal disease opting for upstart PD. In those patients with severe diuretic-resistant heart failure, the surgical placement was contraindicated, or the surgeon was not willing to place the catheter; the patient had severe hypotension and needed circulation support with medication, or the anesthesiologist contraindicated any surgically interventions. Most of patients with ESKD were without prior regularly nephrology care and had eGFR <10 mL/min/1.73 m^2^ but without any sign of uremia, or severe acidosis or high level of potassium. We enrolled those patients with ESKD from our nephrology unit who had to start renal replacement therapy but had no emergencies.

We modified the previously described PDC insertion and reported our method in details before [19]. Briefly, the insertion point for PDC was the left lower quadrant for subsequent intraabdominal placements of the catheter. In our experience, this approach showed the most efficient learning curve for operators, especially in the presence of peritoneal ascites fluid accumulation. We believe this insertion point is much safer, than other insertion points described earlier. We made only a 20 mm length incision to the skin for introducing the catheter. The ascites drainage by physician is a routinely intervention. Moreover, nephrologists place tunneled hemodialysis (HD) catheters routinely, too. The PDC placement was carried out applying a sterile technique and fluoroscopy was used to visualize the advancement of the guidewire. In order to avoid any complications, e.g., injury of abdominal organs, we later used the Veress needle, which is a spring-loaded needle used to create pneumoperitoneum for laparoscopic surgery. Of all the general approaches to laparoscopic access, this technique is the oldest one dating back to 1932 [20]. All patients received prophylactic antibiotics and the procedures were carried out under local anesthesia with 1% lidocaine without epinephrine. We utilized the straight silicone Tenckhoff catheter with two Dacron rings manufactured by Fresenius Medical Care GmbH (Bad Homburg, Germany) in all cases. The PDC was introduced into the peritoneal cavity with the assistance of the COVIDIEN Argyle^TM^ 16 Fr Chronic Catheter Accessory Set (Medtronic, Minneapolis, MN). After we had introduced the catheter, we removed ‘the peel away sheet’. The exit site and the tunnel were made using blunt dissection with a hemostat and created a catheter’s curve in the tunnel. The subcutaneous cuff was placed 2 cm from the skin exit. The Tenckhoff catheter was connected to the dialysis tubing at the end of the procedure, and we checked the drainage with 500 mL dialysis solution.

All patients were started on PD immediately after PDC insertion with a Fresenius Medical Care Stay-safe Balance 1.5% dextrose-based solution (2000 mL, 1.25 mmol/L calcium, 134 mmol/L sodium, 0.5 mmol/L magnesium, 102.5 mmol/L chloride, 35 mmol/L sodium acetate, and 83.2 mmol/L glucose). We started the PD solution exchange two or three times a day in patients with severe heart failure, and the intraperitoneal volume was 2000 mL. The equilibration time was typically 4 or 6 h, depending on the exchange frequency. The basic PD exchange prescription for ESRD’s patients was four exchanges with 2000 mL volume, and 6 h dwell time. All patients were treated with CAPD; we did not use APD, or any other automated equipment. Every PDC insertion was made for hospitalized patients and patients spent at least five days in the hospital after the PDC insertion and initiation of the PD solution exchanges, mostly because of their comorbidities.

## Results

The recruitment period for the procedure was from March 2014 through December 2015 during which time 15 subjects received the described long-term PDC access. The follow-up period lasted until 2016, and patient’s data were collected in 2018.

The patients’ demographic data and major co-morbid conditions are described in Table 1. Their average age was 60 (±18) years; 87% were men and 27% diabetic. Almost half of them (47%; seven of 15) received their PDC for severe diuretic resistant acutely decompensated chronic heart failure and eight (53%) for end-stage kidney disease (ESKD) without heart failure. To be noted, however, a significant part of our cohort (27%) had advanced liver disease. At the end of the clinical follow-up, only three out of the 15 (20%) patients were transferred to HD. We transferred those three patients into HD 1 year after the PDC insertion due to severe peritonitis or ineffective PD treatment. We lost five patients (33%) due to end-stage heart failure, while two (13%) underwent successful cadaveric heart transplantation subsequently (Table 2). Of those five patients four died 16 months after the PDC insertion because of the complications of the end stage heart failure, and one patient died 1 month after PDC insertion due to acute gastrointestinal bleeding. Most of patients in the severe diuretic-resistant heart failure group had ascites. We observed in those patients that the ascites volume had decreased during PD treatment. Anecdotal observations from the survivor cohort suggested a much-decreased rate of repeated hospitalizations with heart failure decompensation in the cardio-renal patients [21]. No serious complications, such as injury to abdominal organs, severe bleeding or infections were observed in any of the cases. There was one patient whose initial PD placement failed due to surgical abdominal adhesions and needed surgical PDC placement. We observed catheter sliding in three of the 15 (20%) patients only. The slipping out occurred on the average one and a half month after the PDC insertion and were thought to be patient behavior-related. One patient lost his PDC due to catheter extrusion and suture dehiscence. We think there is no connection between the catheter lost and PDC insertion, catheter extrusion can occur after surgical insertion, too. We did not observe any early infectious complications; two (13%) patients had late PD-associated peritonitis (Table 3), which had no correlation with PDC insertion. Despite an ‘early’ start on dialysis, i.e., immediately after PDC placement, we experienced PD solution leakage neither during early, nor subsequent follow-ups.

**Table 1.** Demographic data of patients undergoing nephrologist-placed PD catheter insertion.

Demographic data	*n*: 15
Male	13 (87%)
Female	2 (13%)
Age (years)	60 (±18)
Peritoneal dialysis indication	
Heart failure	7 (47%)
End-stage kidney disease	8 (53%)
Comorbid conditions	
Diabetes mellitus	4 (27%)
Hypertension	11 (73%)
Ischemic heart disease	7 (47%)
Liver disease	4 (27%)

**Table 2.** Outcome for patients having undergone percutaneous PD catheter insertion.

Patient outcome	*n*: 15
Died	5 (33%)
Modality change PD/HD	3 (20%)
Heart transplant	2 (13%)
PD ongoing	5 (33%)

**Table 3.** Technical outcomes and complications.

Complications (*n*: 15)	≤1 month	≥1 month
Leakage	0 (0%)	0 (0%)
Catheter loss	1 (6%)	3 (20%)
Drainage disorder	1 (6%)	0 (0%)
Exit site infection	0 (0%)	0 (0%)
Peritonitis	0 (0%)	2 (13%)
Bleeding	0 (0%)	0 (0%)
Injury to abdominal organs	0 (0%)	0 (0%)
Death	0 (0%)	5 (33%)

## Discussion

PDC insertion is primarily performed by surgeons in many medical cultures, including Hungary. The percutaneous blind technique and also the above-described fluoroscopic technique are minimally invasive procedures with decreased surgical risks and the potential for an earlier initiation of an effective PD modality. In our series, an early initiation of renal replacement therapy not only afforded early volume and uremic control, but also prevented large volume ascites accumulation in those with co-morbid advanced heart failure or cirrhosis. Therefore, and only seemingly ‘paradoxically’, initiating the procedure may have contributed to the lack of abdominal fluid leakage in our patients with advanced heart failure and liver cirrhosis. Moreover, PD is an unusual modality, where the efficacy of the procedure is partially disconnected from small solute clearance due to the predominant generation of uremic toxins in the abdominal compartment [22]. The traditional surgical implantation still has its niche when abdominal adhesions are present or when the patient’s excess weight does not permit a safe procedure [23].

The above-described cohort represented the evolution of indications in our center explored initially for those with severe acute decompensated diuretic resistant heart failure [21] and addressing an unmet need for effective volume control. In this patient group, the time spent on PD treatment was an average of 10.8 months and the cause of death remained unrelated to PD. The patients with severe heart failure have a high rate of mortality. The patients in our series were deemed not to be candidates for surgical PDC placement and, not being candidates for cardiac transplantation at that time, only comfort measures could have been offered for them. Our experience herewith and in parts published before [21] suggests that this technique can be performed successfully even in a resource-limited environment and in a low volume setting. Moreover, our series demonstrates success in patients with large degrees of comorbid disease burden and an opportunity to enhance both survival and quality of life. Notwithstanding our positive results, PDC insertion should be performed in medical centers only with both expertise in PD and appropriate surgical background to address procedure-related complications. We believe nephrologist who are familiar with tunneled HD central vein catheter insertion, will able to gain expertise in the PDC technique. For learning a successful technique, however, and minimizing complications with the PDC insertion a formal training at a dedicated center is necessary. We are confident that the modified PDC insertion technique is easier when using the typical abdominal paracentesis entry point. Due to the puncture of the peritoneum and minimizing the incision to the skin, we were able to start PD immediately without waiting for proper wound healing of 6–8 weeks. Notwithstanding, we were using acute PD where immediate catheter use was required.

Peritoneal ultrafiltration can be a therapeutic strategy for patients with severe congestive heart failure (CHF). Peritoneal ultrafiltration is a relatively simple choice for chronic salt and water removal and may be beneficial for the management of patients with CHF who develop severe edema, who are frequently admitted to the hospital and have a much-reduced cardiac reserve [24]. To be noted, we observed the same decreased rate of rehospitalizations in our severe diuretic-resistant heart failure group. During the follow-up period with CAPD in our patients with diuretic-resistant heart failure, we noted an improved quality of life. The readmission rate to hospital dramatically decreased after the start of PD [21]. Thus, of the five patients four died 16 months after the PDC insertion because of complications of end stage heart failure, all patients had superior improvement in quality of life due to PD. We observed that PD-related improvement in left ventricular ejection fraction (LEVF) was associated with better quality of life and reduced hospitalization. Renal dysfunction is prevalent in patients with severe heart failure, and it is an established independent prognostic factor in those patients. Nevertheless, the degree of renal dysfunction did not affect the survival rate, although PD-related improvement in LEVF was associated with better survival [25]. In selected cases, the acute placement of PDCs may be appropriate even in correlation with liver cirrhosis or complex hepato-cardio-renal pathophysiology.

Of note, two of our acutely decompensated chronic heart failure patients received successful orthotopic heart transplants. Our series is the first in Hungary to document acute PD treatment *in lieu* of HD to address diuretic resistant CHF, while also enabling subsequent successful heart transplantation for some of our patients [26]. In our clinical experiment, we were able to treat patients successfully with CAPD in severe chronic heart failure. Our patients on CAPD have been stable and got compensated heart functions. Moreover, our patients with heart failure had improved quality of life till successful orthotopic heart transplantation. Patients with severe diuretics resistant CHF, who require mechanical circulatory support have much higher rates of morbidity and mortality from infections attributable to temporary blood stream access and extracorporeal circulation devices. Accordingly, PD probably should be considered in patients who require renal replacement therapy [27].

## Conclusions

The acute placement of PDC for urgent-start PD can be effectively performed even in a resource-limited environment of a low-volume clinical program. Our results offer a model for minimally invasive, cost-effective integrated care in regional medical centers. The results of our cohort should be interpreted with due consideration of its limitations, limited number of patients undergone PDC insertion, lack of a matched control group, and short period of time during which patients were followed. However, the importance of our investigation is that we could start the PD immediately with modified PDC insertion instead of waiting for 6–8 weeks customary for PDC for chronic use.

## Data Availability

Aggregate patient data are available from the authors upon reasonable request, patients are unidentifiable.
